# Effect of the socket shield technique on alveolar ridge dimensional changes in sites with thin buccal bone phenotype: A randomized clinical trial

**DOI:** 10.4317/medoral.28158

**Published:** 2026-06-28

**Authors:** Luis Miguel Sáez-Alcaide, Carlos Cobo Vázquez, Fabián Pérez González, José González Serrano, Juan López Quiles, Jesús Torres García-Denche

**Affiliations:** 1 Department of Dental Clinical Specialties, Faculty of Dentistry, Complutense University of Madrid, Spain

**Keywords:** Socket shield technique, immediate implant placement, buccal bone thickness, dimensional changes, marginal bone loss.

## Abstract

**Background:**

Post-extraction alveolar ridge remodeling is a major challenge for implant therapy, particularly in sites with a thin buccal bone phenotype. Although immediate implant placement has been proposed to limit ridge resorption, buccal dimensional changes remain frequent. The socket shield technique has been proposed to preserve the periodontal ligament-bundle bone complex and reduce post-extraction bone loss. This randomized clinical trial aimed to compare alveolar ridge dimensional changes following the socket shield technique versus immediate implant placement in sites with buccal bone thickness <1.5mm.

**Materials and Methods:**

This parallel-arm randomized controlled clinical trial included patients requiring extraction of a single-rooted tooth and presenting a thin buccal bone phenotype (BBT <1.5mm). Participants were randomly allocated to receive either socket shield technique (SST) or immediate implant placement (IIP). Horizontal, vertical, and volumetric alveolar ridge changes were assessed using CBCT superimposition at baseline and 1 year. Marginal bone levels were evaluated at implant placement, prosthetic loading, and 1-year follow-up using standardized periapical radiographs. Intergroup comparisons were performed using analysis of covariance (ANCOVA) adjusting for baseline values.

**Results:**

Forty patients completed the study. The SST group demonstrated significantly lower horizontal ridge reduction and buccal vertical bone loss compared with the IIP group at all evaluated levels (p<0.001). Volumetric analysis demonstrated significantly greater overall bone loss in the IIP group (p<0.001). Marginal bone loss did not differ between groups at implant placement; however, significantly greater marginal bone loss progression was observed in the IIP group at prosthetic loading and 1-year follow-up (p<0.001).

**Conclusions:**

In extraction sites presenting a thin buccal bone phenotype, the socket shield technique significantly reduced horizontal and buccal vertical ridge resorption, limited volumetric bone loss, and resulted in improved marginal bone stability compared with immediate implant placement. These findings suggest that SST may represent a predictable strategy for preserving peri-implant hard tissues in anatomically compromised sites.

## Introduction

Tooth extraction is invariably followed by a process of alveolar ridge remodeling, which may compromise subsequent implant placement and esthetic outcomes [1]. This phenomenon is primarily related to the disruption of the bundle bone-periodontal ligament (BB-PDL) complex, as the periodontal ligament plays a fundamental role in maintaining alveolar bone volume and vascularization [2]. Post-extraction remodeling involves dimensional alterations in both the horizontal and vertical planes, with the buccal aspect being particularly susceptible to resorption [3]. These changes represent a major challenge for predictable implant therapy, as they may negatively influence implant positioning, peri-implant tissue stability, and long-term esthetic outcomes, especially in the anterior region [4].

Several alveolar ridge preservation (ARP) strategies have been widely developed to overcome these alterations, including the use of bone grafts, membranes, and minimally invasive extraction techniques [5]. Although ARP procedures have been shown to reduce the magnitude of post-extraction resorption, they are unable to completely prevent the physiologic remodeling process [6]. Similarly, immediate implant placement (IIP) has been advocated as a means to preserve alveolar ridge dimensions; however, available evidence suggests that immediate implant placement alone can only partially compensate for post-extraction bone loss, particularly at the buccal plate [7].

The socket shield technique (SST), first described by Hürzeler *et al*., was introduced as a biologically driven approach designed to preserve the buccal portion of the root together with its periodontal ligament, with the aim to maintain the BB-PDL complex, thereby preserving the vascular supply to the buccal bone and minimizing post-extraction remodeling [8]. Since its introduction, several clinical, radiographic, and histologic studies have reported favorable outcomes associated with SST, including reduced buccal bone resorption and improved peri-implant soft tissue stability [9,10].

Buccal bone thickness (BBT) is a key determinant of post-extraction ridge remodeling, and sites presenting a thin buccal bone phenotype (BBT <1.5mm) are particularly susceptible to pronounced horizontal and vertical resorption [11]. These thin-walled phenotypes represent the most biologically unfavorable conditions for ridge preservation, as both spontaneous healing and IIP provide limited protection against buccal bone collapse [12]. In such critical scenarios, preservation of the periodontal ligament-bundle bone complex may be especially relevant; therefore, techniques designed to maintain the buccal root fragment and its associated vascular supply, such as the SST, may offer greater benefits [13]. Evaluating SST *in site*s with BBT <1.5mm allows assessment of its biological advantages under the most clinically challenging conditions.

Therefore, the aim of this randomized clinical trial was to radiographically evaluate and compare the effect of the socket shield technique and immediate implant placement on horizontal, vertical, and volumetric alveolar ridge dimensional changes at 1-year post-extraction *in site*s presenting a thin buccal bone phenotype (BBT <1.5mm).

## Materials and Methods

*Study design and ethical considerations*

This study was designed as a parallel-arm randomized controlled clinical trial conducted in accordance with the principles of the Declaration of Helsinki. Ethical approval was obtained from the Ethics Committee of Hospital Clínico San Carlos, Madrid, Spain (REF: 14-034; 24 July 2021). The trial protocol was registered at ClinicalTrials.gov (ID: NCT05240417) prior to patient recruitment. Reporting of the study followed the CONSORT guidelines (http://www.consort-statement.org/) for randomized clinical trials.

*Sample size calculation*

The sample size calculation was based on the primary outcome variable, defined as horizontal bone resorption assessed radiographically. Data reported by Li *et al*. in 2024 [14], whose methodological design is comparable to that of the present investigation, were used as a reference. Using G*Power software (version 3.1; Universität Düsseldorf, Germany), an alpha level of 0.05 and a statistical power of 80% were assumed. Based on these parameters, a minimum of 15 patients per group was required, resulting in a total sample size of 30 participants.

*Patient recruitment and study population*

Participants were consecutively recruited from patients attending the Postgraduate Clinic of Oral Surgery at the Complutense University of Madrid (Spain) between January 2022 and October 2024. All eligible individuals received verbal and written information about the study protocol and provided written informed consent prior to enrollment.

*Eligibility criteria*

*Inclusion criteria*

Patients were eligible for inclusion if they met the following criteria:

- Age ≥18 years.

- Indication for extraction of a single-rooted maxillary or mandibular tooth (incisor, canine, or premolar) due to caries, fracture, or prosthetic reasons.

- Presence of at least one adjacent natural tooth mesially and/or distally.

- Presence of a thin buccal bone phenotype, defined as a buccal bone thickness (BBT) <1.5mm, measured on preoperative CBCT.

- Systemically healthy or medically controlled patients classified as ASA I or II.

- Smoking habit of fewer than 10 cigarettes per day

- Good periodontal health, defined as bleeding on probing <20% and plaque index <20%.

*Exclusion criteria*

Patients were excluded if any of the following conditions were present:

- Uncontrolled systemic diseases potentially affecting wound healing (e.g., diabetes mellitus with HbA1c >7%, osteoporosis).

- History of malignancy or previous radiotherapy or chemotherapy.

- Pregnancy, intention to become pregnant, or breastfeeding.

- Acute infection at the extraction site or active periodontal disease.

- Randomization and blinding

After confirmation of eligibility, participants were randomly allocated to one of the two treatment groups by an independent investigator (C.C.V.) who was not involved in the surgical procedures or outcome assessments. Randomization sequences were generated using dedicated software (GraphPad Software Inc., La Jolla, CA, USA). Allocation concealment was ensured throughout the enrollment process. Outcome assessors were blinded to the group assignment.

*Surgical and prosthetic procedures*

All patients underwent a comprehensive preoperative evaluation, including clinical examination, clinical photography, panoramic radiography and CBCT imaging to confirm eligibility. Periodontal status was assessed, and individualized oral hygiene instructions were provided prior to surgery. 

All surgical procedures were performed under local anesthesia by a single experienced oral surgeon (L.S.A.) at the Postgraduate Clinic of Oral Surgery, Complutense University of Madrid. The surgeon did not participate in postoperative assessments.

In the test group, socket shield preparation began with decoronation of the tooth to the level of the gingival margin, then the root was sectioned longitudinally in a mesiodistal direction, and the palatal root fragment was carefully removed while preserving the buccal portion. The remaining buccal root fragment was reduced to the level of the buccal alveolar crest and thinned to approximately 1-1.5mm, ensuring a stable socket shield adjacent to the buccal plate. The absence of mobility of the retained fragment was verified.

In the control group, atraumatic flapless tooth extraction was carried out using forceps and fine elevators, with the aid of a periotome when required.

In both groups, implant osteotomies were prepared to achieve engagement of at least 3-5mm of bone apical to the extraction socket, and implants were placed 1-2mm apical to the buccal alveolar crest. Implants were positioned slightly palatal to maintain a minimum buccal gap of 2mm between the implant and the buccal plate in the control group, as well as between the implant and the socket shield in the experimental group. In the control group, the buccal gap was filled with a bovine cancellous xenograft to prevent spontaneous collapse of the buccal plate, without the use of membranes, whereas no grafting material was used in the experimental sites. In both groups, the socket entrance was sealed with a collagen plug stabilized using a crossed 5/0 polypropylene suture.

Postoperative care was standardized for all patients and included systemic antibiotics (amoxicillin 500 mg three times daily for 5 days), analgesics as required (ibuprofen 600 mg), and twice-daily rinsing with 0.2% chlorhexidine for 10 days. Sutures were removed after 10 days, and postoperative complications were recorded. 

The restorative phase started after a 4-month healing period. Conventional impressions were then taken using open-tray impression posts and silicone material. Two weeks later, a definitive screw-retained zirconia-ceramic crown was delivered and inserted (Figure 1).

*Outcome measures*

- Volumetric changes 

Preoperative cone-beam computed tomography (CBCT1) was performed using a CBCT unit (CS 8100 3D®, Carestream Dental DLL). A second CBCT scan (CBCT2) was obtained 1 year after surgery. All scans were acquired using a standardized protocol with a cylindrical field of view (FOV) of 8 × 8cm and a voxel size of 0.16mm, according to the manufacturer’s recommendations (scan time: 15s; exposure time: 1.8s).

Computer-assisted superimposition of the CBCT datasets was conducted using 3D Slicer® software by an independent, calibrated examiner with more than 5 years of experience in digital DICOM image superimposition (J.T.G.).

Radiographic measurements were conducted following the protocol described by Jung *et al*. [15]. Two independent calibrated examiners performed all measurements (F.P.G. and J.G.S.). Horizontal bone width was assessed by measuring the distance from a vertical reference line to the buccal and lingual bone plates at 1, 3 and 5mm apical to the most coronal aspect of the alveolar crest. Changes in bone width were calculated by subtracting CBCT2 measurements from baseline values, yielding buccal (BW1, BW3, BW5) and lingual (LW1, LW3, LW5) dimensional changes measured at 1, 3, and 5mm below the alveolar crest (Figure 2).

Vertical bone height changes were calculated by measuring the distance from the buccal and lingual bone peaks to a horizontal reference line in both CBCT datasets. Differences between time points represented vertical bone resorption and buccal (BH) and lingual aspect (LH). 

In addition, volumetric bone changes were assessed as a secondary outcome by superimposing STL models generated from CBCT1 and CBCT2 datasets (Figure 3). Absolute and percentage changes were recorded. 

*- *Marginal Bone Level Changes* *

Marginal bone levels around the implants were assessed  at implant placement (T1), at prosthetic loading stage (T2) and at 1-year (T3) of follow-up using standardized sequential periapical radiographs obtained with a long-cone paralleling technique. To ensure reproducible positioning, an individualized occlusal jig with bite registration material (Extrabyte, GC America) was fabricated to standardize the angulation of the radiographic film relative to the X-ray beam. To evaluate the peri-implant bone radiographic changes, interproximal bone levels were measured at mesial and distal sites by measuring the distance from the implant shoulder to the most coronal point of bone-to-implant contact and the average value was obtained (Figure 4).

*Statistical analysis*

Statistical analyses were performed using SPSS software (version 28.0; IBM Corp., Chicago, IL, USA). Continuous variables were analyzed using analysis of covariance (ANCOVA) to compare dimensional changes between treatment groups while controlling for baseline values. 

Measurement reliability was assessed using the intraclass correlation coefficient (ICC) with 95% confidence intervals. Statistical significance was set at α=0.05.

## Results

*Study sample and data distribution*

A total of 50 patients were assessed for eligibility. After screening, seven individuals were excluded (six did not fulfill the inclusion criteria and one declined participation). The remaining 43 patients were randomized into two groups: 22 patients were allocated to the experimental group (SST) and 21 to the control group (IIP). During the follow-up period, three patients were lost for personal reasons (two in the SST group and one in the IIP group). Consequently, 40 patients (17 men and 23 women) with a mean age of 56.05 years completed the study Supplementary 1 (https://www.medicinaoral.com/medoralfree01/28158/Suppl1.pdf).

All included participants were systemically healthy, and no intraoperative or postoperative complications were observed throughout the study period. Baseline demographic and clinical variables, including age, sex, smoking habits, tooth location, reason for extraction, and buccal bone thickness (BBT), were comparable between groups, with no statistically significant differences detected (Table 1).

*Bone dimensional changes*

Superimposition of baseline and 1-year CBCT datasets revealed significantly smaller alveolar ridge dimensional changes in the SST group compared with the IIP group. Horizontal ridge width reduction measured at 1, 3, and 5mm below to the alveolar crest averaged 0.09, 0.09, and 0.13mm, respectively, in the SST group, whereas corresponding values of 0.87, 0.75, and 0.71mm were observed in the IIP group.

Evaluation of vertical bone dimensional changes showed a mean buccal height (BH) reduction of 0.10mm and a lingual height (LH) reduction of 0.51mm in the SST group, compared with mean reductions of 1.03mm (BH) and 1.02mm (LH) in the IIP group. Although differences at the lingual aspect were not statistically significant, the SST group consistently exhibited lower vertical and horizontal dimensional changes across all assessed parameters.

Intergroup comparisons revealed statistically significant differences in favor of SST for buccal height reduction (0.94mm; 12.39%; *p<*0.001) and buccal bone width reduction at 1mm (0.78mm; 9.68%; *p<*0.001), 3mm (0.66mm; 7.35%; *p<*0.001), and 5mm (0.58mm; 5.32%; *p<*0.001). Additionally, volumetric analysis demonstrated a significantly lower bone resorption in the SST group, with a mean intergroup difference of 30.57mm³ (*p<*0.001) (Table 2).

*Marginal Bone Loss*

No statistically significant differences in marginal bone loss were detected between groups at the early evaluation time point (T1; *p=*0.971). In contrast, at subsequent follow-up assessments (T2 and T3), marginal bone loss was significantly greater in the control group (both *p<*0.001). Longitudinal analysis of marginal bone level changes demonstrated significantly larger progression of bone loss in the control group across all assessed time intervals (T1-T2, T1-T3, and T2-T3; all *p*<0.001) (Table 3).

## Discussion

The present randomized clinical trial evaluated horizontal, vertical, and volumetric alveolar ridge changes following tooth extraction by comparing immediate implant placement and the socket shield technique *in site*s presenting a thin buccal bone phenotype (BBT <1.5mm). This anatomically unfavorable condition is widely recognized as being associated with an increased risk of post-extraction ridge collapse [16]. Within this context, the results demonstrated that SST provided a significant protective effect against alveolar bone resorption compared with IIP, particularly at the horizontal and buccal vertical dimensions.

One of the most clinically relevant findings was the significantly greater horizontal resorption observed in the IIP group compared with the SST group at all evaluated levels (1, 3, and 5mm apical to the crest). Consistently, vertical dimensional changes analysis revealed greater buccal height loss in the IIP group, whereas lingual height changes did not differ significantly between groups. Volumetric assessment further corroborated these findings, demonstrating significantly greater overall bone loss in the IIP group. 

Beyond their statistical significance, the magnitude of the observed dimensional changes should also be considered from a clinical perspective. Differences in the range of millimeters at the buccal aspect may have a direct impact on peri-implant contour stability, particularly in aesthetically demanding regions and in patients presenting with a thin buccal bone phenotype. Even limited buccal bone reduction may translate into soft tissue collapse and apical displacement of the midfacial mucosal margin, thereby compromising the final aesthetic outcome. Therefore, the reduced buccal resorption observed in the SST group may be clinically relevant in terms of preserving peri-implant contour and supporting soft tissue stability.

From a biological perspective, preservation of the buccal root fragment may attenuate ridge collapse by maintaining the periodontal ligament-bundle bone complex and its vascular supply, thereby reducing buccal bone resorption [17]. Buccal bone thickness (BBT) has been consistently identified as a critical factor influencing post-extraction ridge remodeling, particularly in the anterior maxilla, where most sites present a thin buccal plate [18]. Sites with BBT <1.5mm are especially susceptible to pronounced horizontal and vertical resorption, and represent the most challenging clinical scenarios, in which conventional extraction or immediate implant placement alone may be insufficient to prevent significant dimensional alterations [19].

In this context, the results of the present study indicate that the SST may be particularly advantageous in anatomically unfavorable sites.

The findings of the present study are consistent with current clinical and radiographic evidence supporting the socket shield concept. Using an evaluation protocol comparable to that of the present investigation, Li *et al*. reported improved and more stable bone preservation with SST compared with IIP after a 5-year follow-up [14]. Similarly, in a recent randomized clinical trial, Liao *et al*. observed that SST was more effective than IIP alone in maintaining the facial peri-implant tissue profile [20]. In addition, a recent systematic review and network meta-analysis concluded that implant-supported rehabilitations performed with SST were associated with superior esthetic outcomes and more effective preservation of both hard and soft tissues when compared with IIP, suggesting that it is an optimal approach for esthetically demanding sites [21]. Overall, the available evidence reinforces the biological rationale and clinical relevance of preserving the buccal root fragment.

Analysis of marginal bone loss revealed a significantly greater progression of bone loss in the IIP group at intermediate and final follow-up periods. In contrast, sites treated with SST exhibited greater marginal bone stability over time, indicating that the benefits of ridge preservation may extend beyond the early post-extraction phase. Longitudinal analysis further demonstrated significantly greater marginal bone loss in the control group across all evaluated intervals (T1-T2, T2-T3, and T1-T3). These findings suggest that the protective effect of the socket shield technique becomes more apparent over time, potentially related to sustained preservation of the buccal periodontal ligament and reduced peri-implant bone remodeling. The reported MBL changes by other studies performing SST with similar protocols range from 0.08±0.14mm in [22] to 0.54±0.06mm [23], which are comparable to the results obtained in the present study. Similarly, MBL changes at IIP in our study are similar to those at reported in other studies, which range from 0.71±0.68mm [24] to 1.21±1.44mm [25]. It should be noted that most studies reporting marginal bone loss around SST and IIP do not exclusively include sites with thin buccal bone phenotypes, which may partly explain the marginal bone loss values observed in the present study.

Several limitations should be acknowledged. The sample size was relatively limited and the follow-up period, although clinically relevant, does not allow conclusions regarding long-term stability. Furthermore, the study focused exclusively on thin buccal bone phenotypes, which may limit the generalizability of the findings to sites with thicker buccal plates. An important aspect to consider when interpreting the present findings is that the two groups were not strictly identical in terms of adjunctive procedures. In the control group, the buccal gap was grafted with bovine xenograft, whereas no grafting material was used in the SST group. This difference may have influenced the dimensional outcomes and should be taken into account when attributing the observed effects. Another relevant aspect when interpreting the present findings is the absence of soft tissue, aesthetic, and patient-reported outcome measures. While the primary objective of this study was focused on hard tissue dimensional changes, it is well established that the maintenance of peri-implant aesthetics is critically dependent on the integrity of the buccal cortical plate. Therefore, the reduced buccal resorption observed in the SST group may have relevant implications for the preservation of peri-implant aesthetics. However, since parameters such as the Pink Esthetic Score (PES) or patient-reported outcome measures were not assessed, no direct conclusions can be drawn regarding the aesthetic performance of the techniques. All procedures were performed by an experienced operator in order to ensure standardization and minimize variability. However, this may limit the external validity of the findings, as such conditions may not fully reflect routine clinical practice. In addition, the socket shield technique is inherently technique-sensitive, and clinical outcomes may depend on the operator’s level of experience.

Therefore, future studies with larger cohorts, longer follow-up periods, and combined hard and soft tissue assessments are warranted. Within these limitations, the results of the present study indicate that the SST may contribute to the preservation of buccal alveolar bone dimensions and improved marginal bone stability in extraction sites with a thin buccal bone plate. Within the limitations of the present study, the socket shield technique demonstrated a clear protective effect against buccal alveolar bone resorption in anatomically unfavorable sites characterized by a thin buccal bone phenotype.

## Conclusions

The socket shield technique may represent a valuable treatment option for ridge preservation *in site*s with a thin buccal bone phenotype (<1.5mm), as it was associated with reduced horizontal and buccal vertical bone loss, lower volumetric resorption, and improved marginal bone stability compared with conventional immediate implant placement. However, these findings should be interpreted within the limitations of the study and further long-term investigations are required to confirm these results. 

## Supplementary Material

Supplementary material 1

## Figures and Tables

**Table 1:** Table 1: Demographic data of the included patients.

**Baseline characteristics**	**Control Group**	**Experimental Group**
Age (years)	56.81 (8.43)	55.3 (12.19)
Male/Female	8/12	9/11
Smokers	4	3
Maxilla/Mandible	16/4	15/5
Incisors/Canines/Premolars	11/1/08	10/1/09
Reason for extraction (caries/endodontic failure/fracture/prosthetic reasons)	9/2/2/7	9/2/3/6
BBT 1mm below crest	0.70±0.17mm	0.78±0.19mm
BBT 3mm below crest	0.79±0.15mm	0.91±0.21mm
BBT 5mm below crest	1.01±0.14mm	1.08±0.22mm

BBT: Buccal bone thickness

**Table 2:** Table 2: Mean dimensional changes between the baseline and the 1-year follow-up.

**Outcome**	**Group**	**N**	**Mean mm (%)**	**SD**	**Medians**	**Significance** ^a^
BW1	Test	20	0.095 (1.14±0.51)	0.04	0.09	<0.001
	Control	20	0.871 (10.82±4.64)	0.24	0.86	
BW3	Test	20	0.090 (1.02±0.44)	0.03	0.10	<0.001
	Control	20	0.752 (8.37±3.51)	0.31	0.68	
BW5	Test	20	0.136 (1.87±0.61)	0.08	0.13	<0.001
	Control	20	0.718 (7.19±3.08)	0.15	0.74	
LW1	Test	20	0.252 (2.96±0.89)	0.05	0.26	0.009
	Control	20	0.509 (6.63±2.34)	0.17	0.50	
LW3	Test	20	0.269 (2.91±0.69)	0.05	0.27	0.015
	Control	20	0.476 (5.68±4.01)	0.22	0.40	
LW5	Test	20	0.231(2.34±0.88)	0.08	0.24	0.075
	Control	20	0.436 (4.95±1.98)	0.20	0.50	
BH	Test	20	0.103 (1.23%±0.75)	0.06	0.11	<0.001
	Control	20	1.038 (13.62%±4.32)	0.28	1.06	
LH	Test	20	0.512 (11.59±3.59)	0.24	0.95	0.247
	Control	20	1.029 (12.72±5.251)	0.36	1.07	
Volume	Test	20	4.95mm^3^	1.56	4.73	<0.001
	Control	20	35.52mm^3^	12.04	31.49	

SD: Standard deviation; a: The significance level is 0.050; BW1: Buccal bone width at 1mm; BW3: Buccal bone width at 3mm; BW5: Buccal bone width at 5mm; LW1: Lingual/palatal bone width at 1mm; LW3: Lingual/palatal bone width at 3mm; LW5: Lingual/palatal bone width at 5mm; BH: Buccal height; LH: Lingual height.

**Table 3:** Table 3: Mean marginal bone loss changes at different follow-up time points.

**MBL**	**Group**	**Mean mm**	**SD**	**Medians**	**Significance** ^a^
T1 (surgery)	Test	-0.054	0.01	-0.06	0.971
	Control	-0.052	0.01	-0.06	
T2 (prosthetic delivery)	Test	0.350	0.07	0.36	<0.001
	Control	0.804	0.11	0.81	
T3 (1 year)	Test	0.418	0.07	0.42	<0.001
	Control	1.085	0.14	1.10	
MBL Changes T1-T2	Test	0.296	0.06	0.29	<0.001
	Control	0.751	0.11	0.76	
MBL Changes T1-T3	Test	0.364	0.06	0.35	<0.001
	Control	1.032	0.14	1.04	
MBL Changes T2-T3	Test	0.068	0.01	0.07	<0.001
	Control	0.281	0.05	0.29	

MBL: Marginal bone loss; SD: Standard deviation; a: Significance level: 0.05.

**Figure 1:**
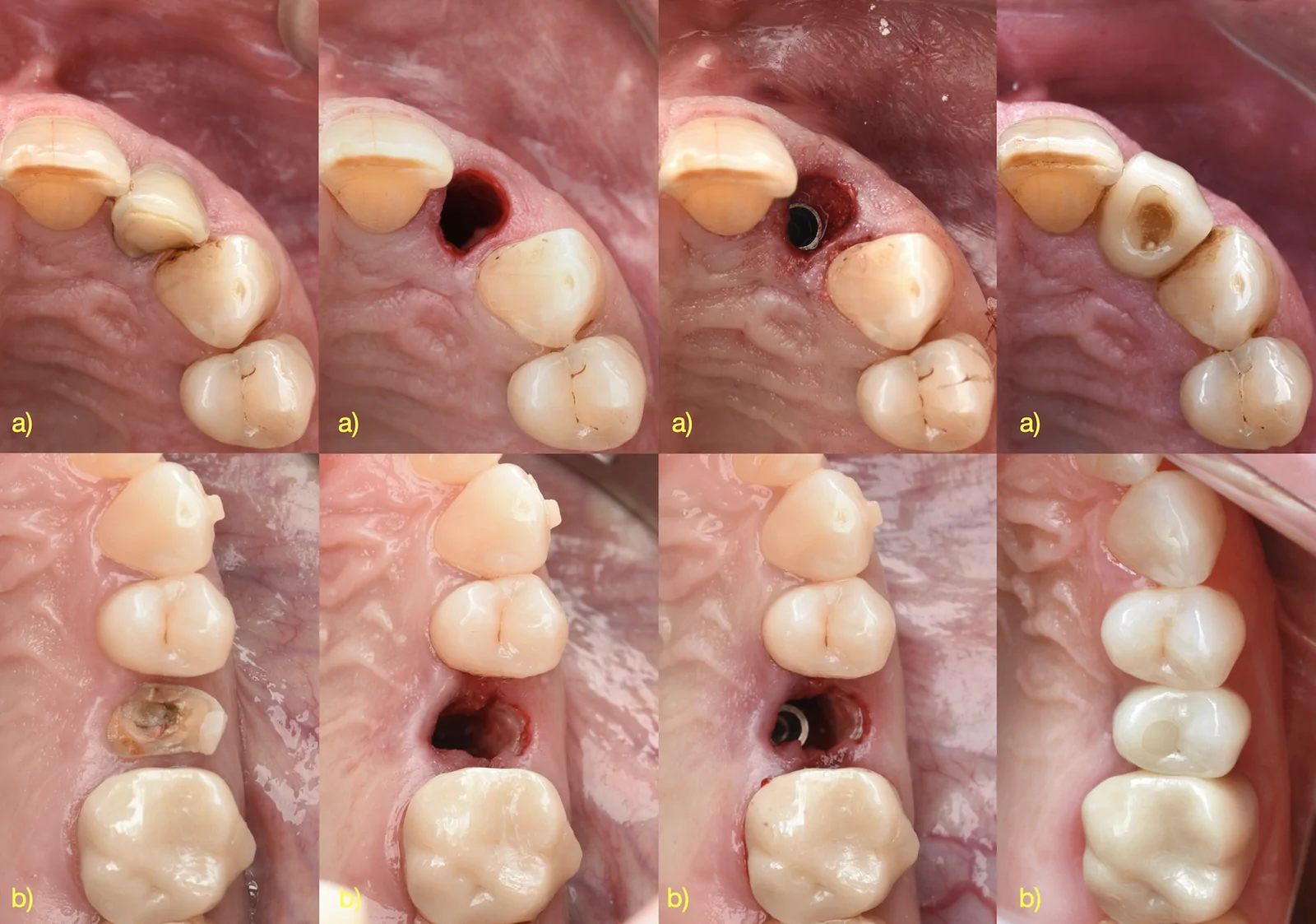
Fig. 1: Step by step description of the treatment procedures: a) control group; b) experimental group.

**Figure 2:**
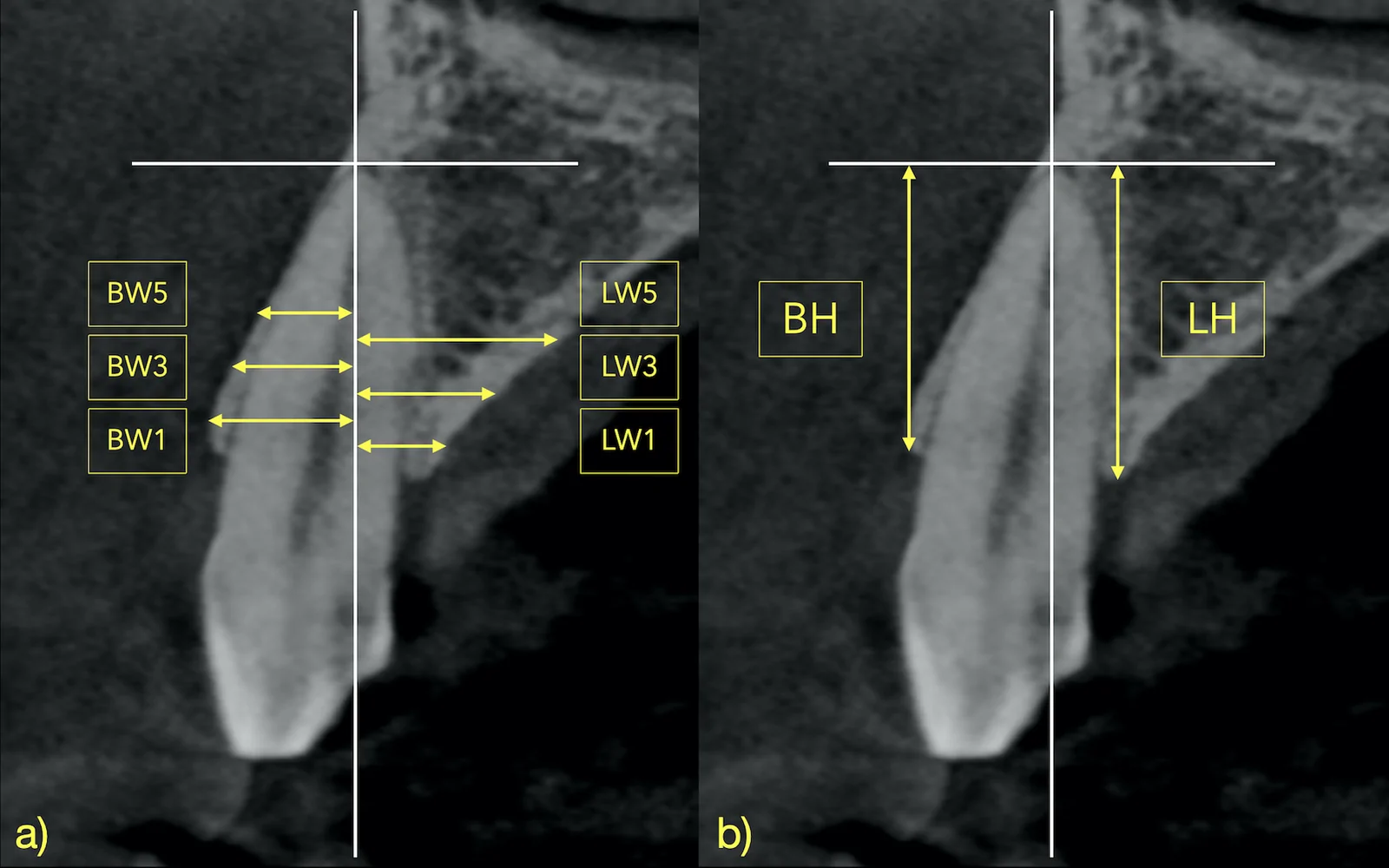
Fig. 2: Radiographic measurements on CBCT: a) Horizontal references for changes in buccal and lingual width at 1, 3 and 5mm; b) Vertical references of the buccal and lingual plate height.

**Figure 3:**
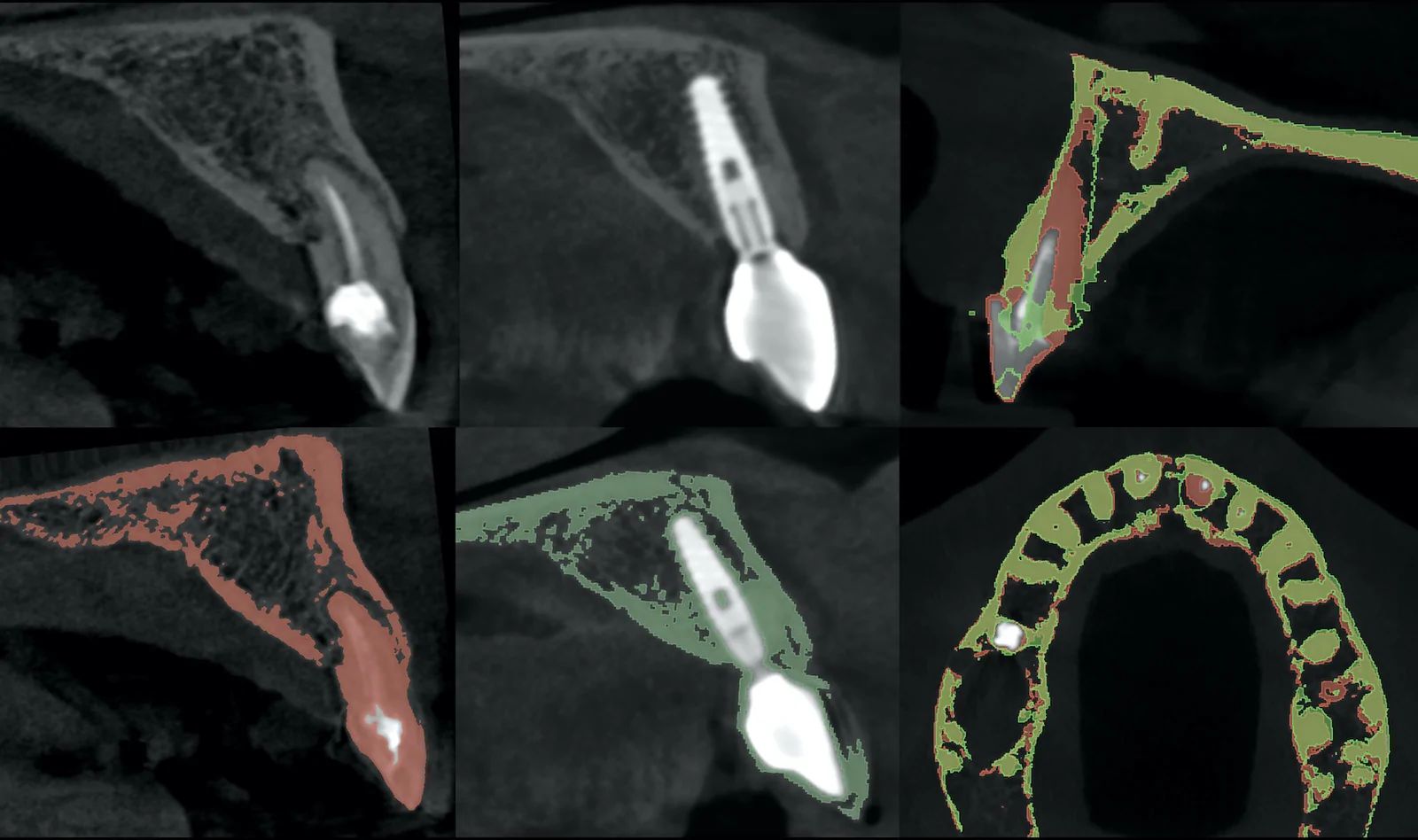
Fig. 3: Superimposition of CBCT1 and CBCT2.

**Figure 4:**
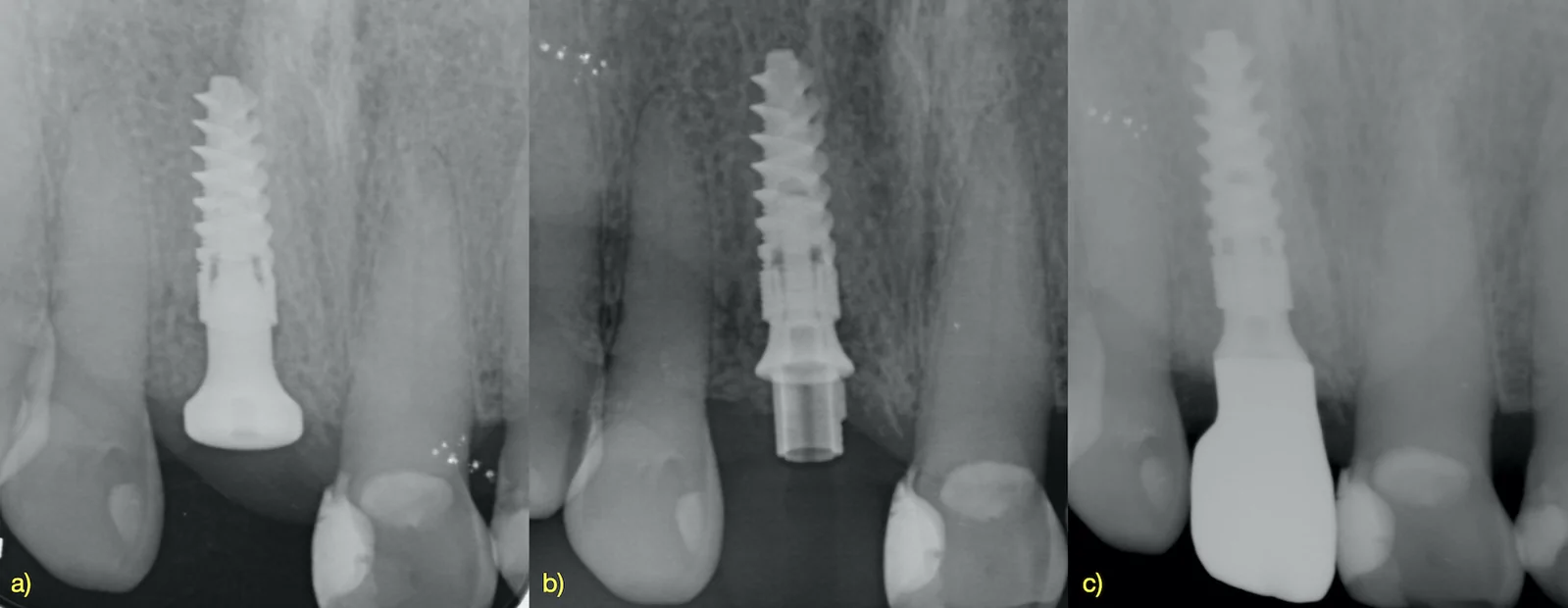
Fig. 4: Periapical radiograph at: a) Implant placement (T1); b) Prosthetic loading stage (T2); c) 1-year follow-up (T3).

## Data Availability

None.
