# Supplementary material for: Effect of the socket shield technique on alveolar ridge dimensional changes in sites with thin buccal bone phenotype: A randomized clinical trial
Source: Med Oral Patol Oral Cir Bucal. 2026 Jun 28;31(5):e707–15. doi: 10.4317/medoral.28158 (PMC13636453; doi:10.4317/medoral.28158)
Supplement: Supplementary file 1 [file medoral-31-05-e707-s001.pdf]

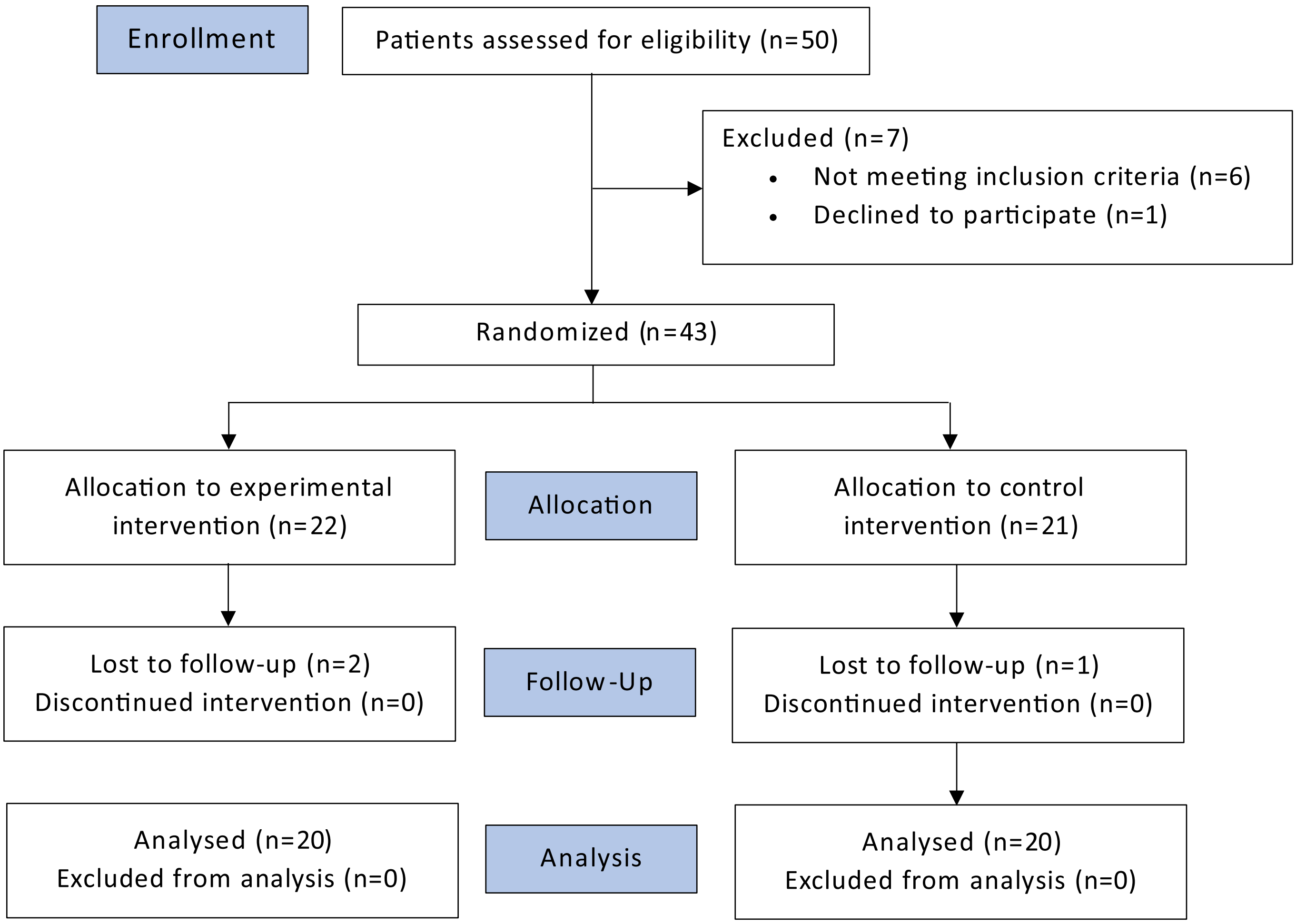

Flowchart diagram of the study protocol according to Consort guidelines.
